# Atomically Thin Current Pathways in Graphene through Kekulé-O Engineering

**DOI:** 10.1021/acs.nanolett.3c04703

**Published:** 2024-02-08

**Authors:** Santiago Galván y García, Yonatan Betancur-Ocampo, Francisco Sánchez-Ochoa, Thomas Stegmann

**Affiliations:** † Instituto de Ciencias Físicas, Universidad Nacional Autónoma de México, 62210 Cuernavaca, México; ‡ Instituto de Física, Universidad Nacional Autónoma de México, 04510 Ciudad de México, México

**Keywords:** quantum transport, graphene, Kekulé distortions, solitons, Jackiw−Rebbi model

## Abstract

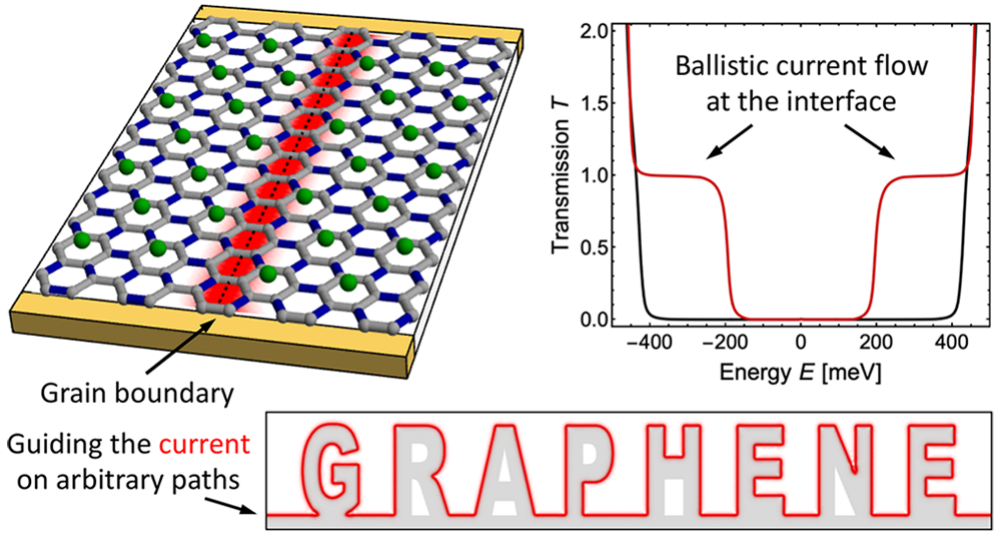

We demonstrate that the current flow in graphene can be guided on atomically thin current pathways by the engineering of Kekulé-O distortions. A grain boundary in these distortions separates the system into topologically distinct regions and induces a ballistic domain-wall state. The state is independent of the orientation of the grain boundary with respect to the graphene sublattice and permits guiding the current on arbitrary paths. As the state is gapped, the current flow can be switched by electrostatic gates. Our findings are explained by a generalization of the Jackiw–Rebbi model, where the electrons behave in one region of the system as Fermions with an effective complex mass, making the device not only promising for technological applications but also a test-ground for concepts from high-energy physics. An atomic model supported by DFT calculations demonstrates that the system can be realized by decorating graphene with Ti atoms.

Controlling and steering the current flow at the nanoscale are ongoing problems in science and engineering because they are of vast importance for all nanoelectronic devices. One of the most successful strategies has been the electrostatic gating of semiconductors, which allows switching on and off the current flow and eventually enables devices like field effect transistors which operate in all computer chips. However, further miniaturization of these devices is approaching its end, making it necessary to investigate new methods and materials.

Such a new way to control the current flow is offered by topological insulators, where the bulk is an insulator while the edges feature conducting states which are topologically protected and can be used to guide efficiently the current.^1−3^ Due to this phenomenal property, topological insulators have become a hot-topic in solid-state research, and their discovery has been awarded with the Nobel prize in 2016.^4^ In the search of a new material capable of replacing silicon in nanoelectronic devices, graphene will certainly come to mind, given its exceptional transport properties.^5−7^ However, the absence of a band gap in graphene makes it impossible to switch off the current flow. Even worse, pristine graphene is not topological (as it is gapless) and the Klein tunneling due to the pseudospin of the electrons in graphene prevents any confining and guiding of the current flow through electric gates.^8−11^ Several strategies have been discussed how to open a band gap in graphene. For example, narrow nanoribbons show a band gap but are difficult to fabricate although impressive advances have been made by chemical synthesis.^12−16^ Other 2D materials with an intrinsic band gap like the transition metal dichalcogenides (TMDs) or phosphorene suffer from low electron mobility or rapid degradation of the material.^17,18^

A recently discussed possibility to alter the properties of graphene is through Kekulé distortions where—inspired by the ideas of August Kekulé for the benzene molecule^19^—the carbon bonds are altered periodically.^20^ It has been shown that graphene on a Cu(111) substrate shows Y-shaped bond alternations and is named therefore Kekulé-Y graphene.^21^ In this case, the Dirac cones are mapped to the Γ point and remain gapless but can have different Fermi velocities that can be employed to induce birefringence in a spherical pn junction.^22,23^ In order to open a gap in Kekulé-Y graphene, it has been proposed using Kekulé-Y bilayers or on-site potentials.^24,25^ Another Kekulé distortion is a benzene-ring-like bond texture named Kekulé-O graphene^26−28^ that was recently observed in graphene deposited in SiC with Li intercalations.^29^ In this case the electronic structure possesses a band gap proportional to the degree of deformation.^30^

In this Letter, we propose a device where the current can be guided on arbitrary atomically thin pathways through the system and additionally switched by electric gates. The current pathways are generated through the engineering of Kekulé-O distortions with a grain boundary, which separate the system into two distinct regions, see Figure 1. We show that a ballistic domain wall state, named also soliton, arises at the interface of these regions. Our work is a realization (and generalization) of the seminal work by Jackiw and Rebbi,^31^ who showed in the context of high-energy physics that a soliton exists at the interface of two topologically distinct regions. Much later the existence of this soliton was demonstrated for the (at that time) newly discovered class of topological insulators.^32^ Semenoff and co-workers suggested the realization of the soliton in a graphene heterojunction composed of two parts with different staggered potentials.^33^ More recently, implementations on the basis of polariton graphene^34^ and even on the basis of Kekulé-O graphene have been proposed.^35−38^ The soliton has not yet been observed directly in graphene but in emulation experiments like photonic crystals^39,40^ and acoustic resonator networks.^41^ Our paper goes beyond these works in the following way: The soliton arises due to a grain boundary between two regions of Kekulé-O distorted graphene and does not rely on differently modified bonds in the two regions (or on-site potentials). Most importantly, as realizations of Kekulé-O graphene are still rare, we demonstrate theoretically that our system can be realized by decorations of graphene with Ti atoms. In our case, the soliton has a gap and can therefore be switched efficiently by gates. Moreover, it does not depend on the orientation of the graphene sublattice, allowing the electron current to be guided on arbitrary paths by means of suitably engineered grain boundaries. Another more subtle finding is that fact that the electrons in our system behave as a relativistic particles with a *complex* effective mass, making our system not only promising for technological applications but also a test-ground for concepts from high-energy physics. All our findings are supported by numeric calculations as well as analytical results based on an effective low-energy Hamiltonian.

**Figure 1:**
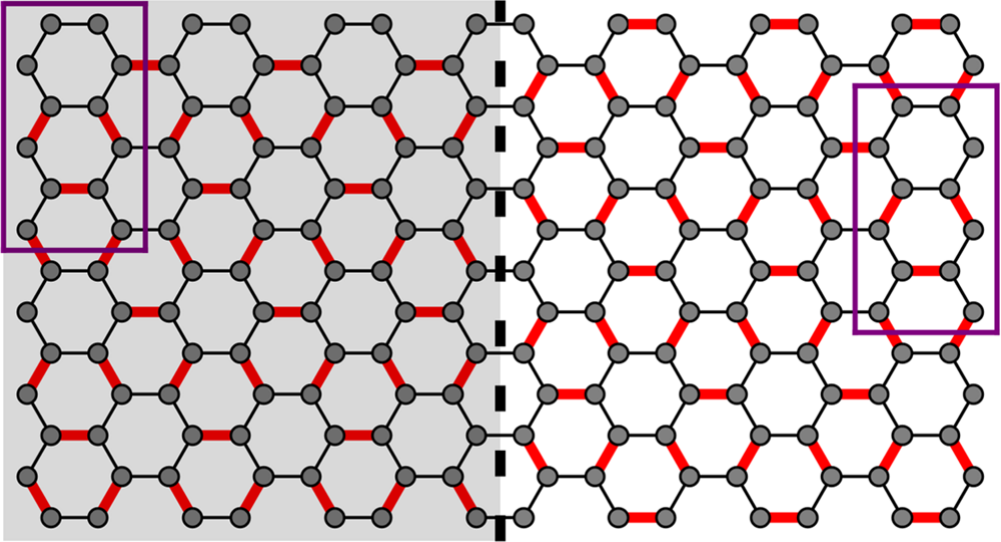
Sketch of the studied system, graphene with Kekulé-O bond distortions where the red colored bonds are strengthened by Δ. A grain boundary separates the system into two regions; see gray and white background color, the displacement between the atoms in the purple rectangle, and the black dashed vertical line.

We consider a sheet of graphene where certain carbon bonds are changed; see the red bonds in Figure 1. These changes are in analogy to the Kekulé model for the benzene molecule and therefore, the material is named Kekulé-O (Kek-O) graphene. The system is separated into two regions by a grain boundary, see the vertical displacement of the unit cell framed by the purple rectangle. The position of the grain boundary is indicated by the black dashed vertical line.

The system is modeled by a nearest neighbor tight-binding Hamiltonian, introduced by Gamayun et al.^22^
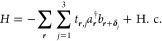
1where *a*^†^ is the creation operator for electrons on graphene’s sublattice  and *b* the annihilation operator on sublattice . The vectors **δ**_*j*_ point to the three nearest neighboring carbon atoms. The Kekulé distortions of the carbon bonds are taken into account by
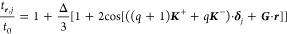
2where *t*_0_ ≈ 2.8 eV is the unmodified bond with a distance *d*_0_ ≈ 0.142 nm and Δ its distortion (measured in multiples of *t*_0_). The vectors ***K***^±^ are the high symmetry points and ***G*** their difference. The parameter *q* is an integer number with value 1 and 0 in the left and right region of Figure 1, respectively.

Making a Taylor expansion to first order around the Γ point, we obtain the effective Hamiltonian of Kek-O graphene
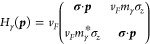
3where the Fermi velocity and the effective electron mass are given by
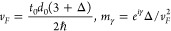
4with the definition . We see that Δ gives a finite mass to the electrons while the parameter *q* enters in a complex phase. Inserting the two possible values of *q*, we obtain in the left region a positive mass, *m*_γ=0_ = Δ/*v*_*F*_^2^, while in the right region a *complex* effective mass is assigned to the electrons, . Note that the inclusion of a complex mass leaves unaffected the hermiticity of the Hamiltonian, and the phase 2π/3 represents the rotation of the distorted benzene rings in the left region with respect to the right region. The energy bands of this Hamiltonian
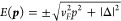
5show the typical dispersion of massive Dirac Fermions with a band gap of size 2|Δ|. The above energy bands are 2-fold degenerate due to the valley degree of freedom. They do not depend on the parameter γ, because for a homogeneous system we can get rid of the complex phase in the mass by a gauge transformation. Note that in other work,^35−38^ different bond modifications are used where in the left region the bonds are strengthened (*t*_0_ + Δ) while in the right region they are weakened (*t*_0_ – Δ), which leads to a negative effective electron mass in the right region and a gapless soliton.

We investigate the electronic transport in the system and analyze the possibility of steering the current flow on arbitrary atomically thin pathways. For that, further theory to understand our findings is developed. In the following, a bond modification Δ = 0.15*t*_0_ ≈ 420 meV is used. In Figure 2, we show the local density of states (a) and the local current (b) calculated by means of the Green’s function method (see the Supporting Information for details). The electrons have energy *E* = 0.5Δ and are injected at the bottom edge. We observe the localization of a domain wall state and a ballistic current at the interface of the grain boundary (dashed black line), which separates the system into two regions (gray and white shaded regions). Note that the LDOS also shows the states induced by the metallic contacts (described by a wideband model) at the bottom and top system edges. Figure 2(c) displays the transmission between the two contacts, studying in this case a smaller system with semi-infinite leads in order to avoid back-reflections at the system edges. It confirms a ballistic state, *T* = 1, within the energy range Δ/2 ≲ |*E*| ≲ Δ(red curve). For comparison, we also show the transmission for a system without a grain boundary (black curve), where this ballistic state is absent. The quantization of the transmission at *T* = 1 can be understood by the fact that, despite the existence of two degenerate valleys in Kek-O graphene, only a single soliton arises at the grain boundary, see eq 9 and its discussion below. We also added to Figure 2 the transmission for a system consisting of two regions of Kek-O graphene without a grain boundary, which are separated by a small ribbon of pristine graphene (about 8 carbon rings wide), effectively forming a quantum well (blue curve). In this case, we observe a *T* = 2 quantization in a certain energy range, which can be distinguished clearly from the *T* = 1 for the soliton and is due to a valley-degenerate ballistic state confined in the quantum well. In the Supporting Information, we demonstrate that the soliton state is rather robust against defects and imperfections.

**Figure 2:**
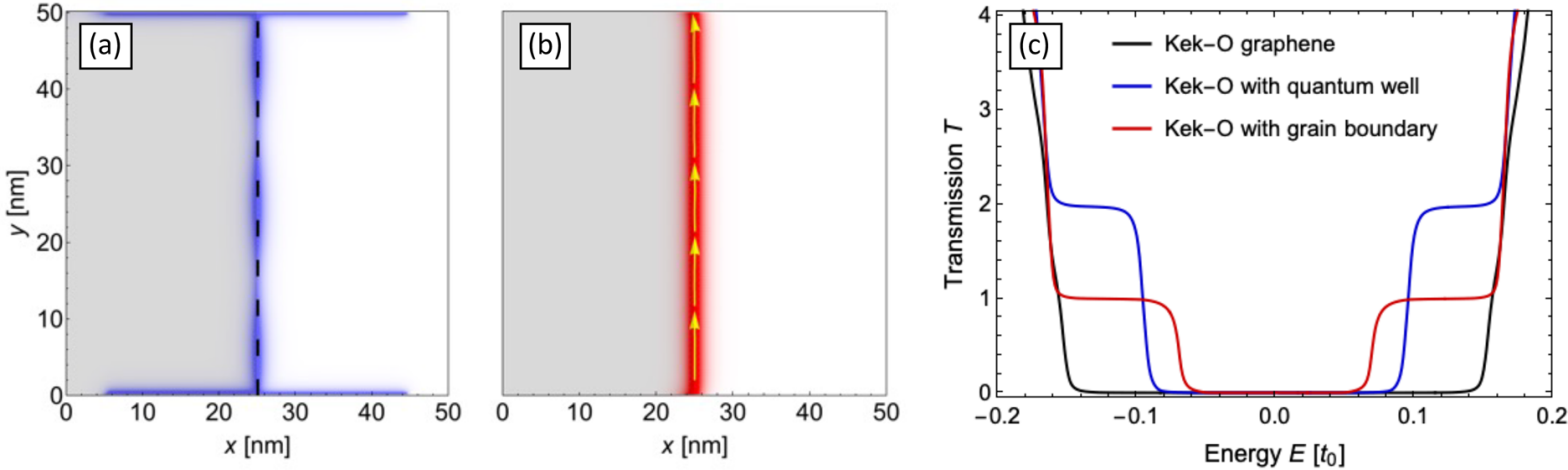
Local density of states (a, blue color shading) and local current (b, red color shading and yellow arrows) for electrons with energy *E* = 0.5Δ ≈ 210 meV injected at the bottom edge. A bond modification of Δ = 0.15*t*_0_ is used. A domain wall state is observed clearly at the interface of the two regions (black dashed line). The transmission between the top and bottom contacts (c, red curve) confirms this ballistic state (*T* = 1) within the energy range Δ/2 ≲ |*E*| ≲ Δ. This state is absent in a system without a grain boundary (black curve). In the case of a quantum well (blue curve), formed by stripe of pristine graphene between two regions of Kek-O graphene (without grain boundary), we find *T* = 2 in a finite energy range.

As shown in Figure 3, the ballistic state is independent of the orientation of the graphene sublattice with respect to the grain boundary. Even complex current flow patterns can be generated by a suitably shaped grain boundaries, like the name of this journal. This figure also shows that the precise bond modifications at the interface are not important but the global existence of the grain boundary between the two regions, making them topological distinct.

**Figure 3:**
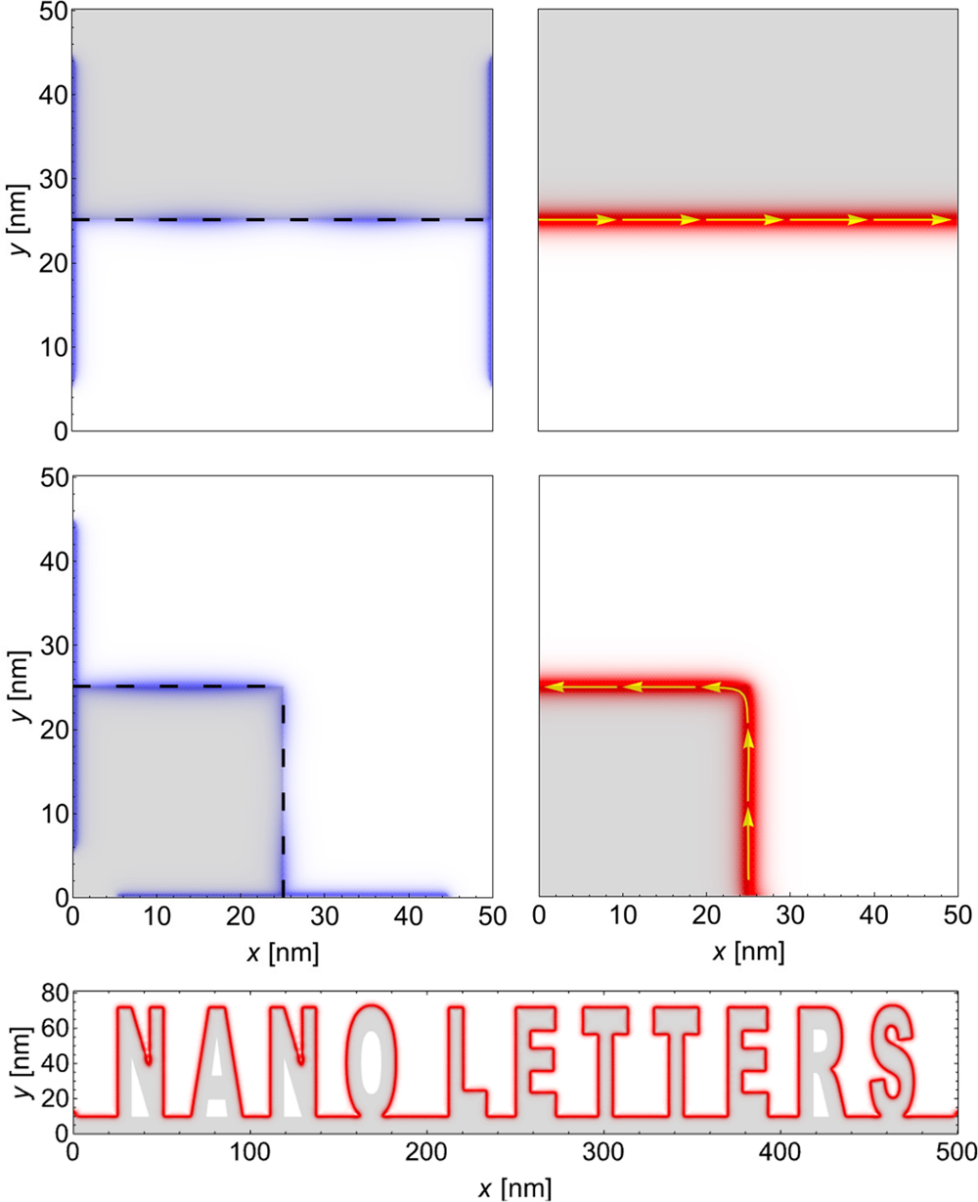
Soliton does not depend on the orientation of the graphene sublattice and therefore can follow a corner or even more complex patterns, like the name of this journal.

In the following, we will show that our findings can be explained by a generalized Jackiw–Rebbi model, that was originally developed to explain the emergence of a soliton at the interface between two regions, where in one of them the electrons have a positive effective mass and a negative mass in the other.^31^ Here, we generalize this model by permitting a complex valued effective electron mass through the parameter γ. The model consists in finding the domain wall state through the following Ansatz
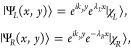
6where |χ_*L*/*R*_⟩ are the four-component spinors in the left and right region, respectively. We assume that this state propagates along the *y* direction and decays exponentially in the *x* direction. This Ansatz has to fulfill the Schrödinger equations in the two regions
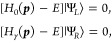
7which allow us to calculate the depth lengths
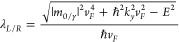
8From the continuity of the wave function at the interface, |Ψ_*L*_(*x* = 0, *y*)⟩ = |Ψ_*R*_(*x* = 0, *y*)⟩, we obtain finally the energy bands of the soliton
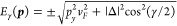
9

In Figure 4(a), we show this energy band (red dashed curve) together with the energy band from eq 5 for Kek-O graphene without a grain boundary (green dashed curve). The solid blue curves are the numerically calculated energy bands of a finite nanoribbon of Kek-O graphene with grain boundary. Details of the calculations can be found in the Supporting Information. The analytical models agree perfectly with the numerically calculated energy bands and also match the transmission plateaus shown in Figure 2.

**Figure 4:**
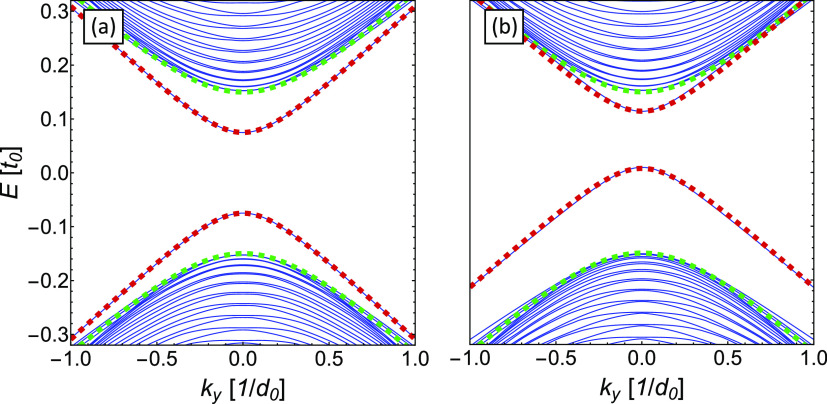
Band structure of the system. (a) The dashed red curve shows the energy band of the soliton from eq 9, while the green dashed curve shows the energy bands of Kek-O graphene without a grain boundary from eq 5. The solid blue curves indicate the numerically calculated bandstructure. (b) An on-site potential ε = 0.3*t*_0_ at the interface moves the soliton bands to higher energies and reduces the band gap, as observed in the DFT calculations of graphene with Ti adatoms (see Figure 5 below).

The energy band of the soliton shows a gap of size 2|Δ| cos(γ/2), which can be modulated through the parameter γ. In the case of Kek-O graphene with a grain boundary, we have γ = 2π/3 and observe that the gap of the soliton |Δ| is half of the bulk value, in perfect agreement with the transmission curve in Figure 2. The system of Kek-O graphene where a grain boundary is absent but the bonds in the right region are weakened by Δ^35−38^ can be represented by γ = π and therefore leads to a gapless edge state.

We propose to realize the system by decorating graphene periodically with Ti atoms on hollow sites of the carbon rings to perturb the carbon bonds and generate a superlattice with fused Kek-O segments. The atomic model can be seen in Figure 5(c,d), where the Ti atoms (green spheres) are absorbed on graphene. A grain boundary in the graphene superlattice is generated by the displacement of the Ti atoms. Note that here, we implement two grain boundaries as we use periodic boundary conditions in both the *x* and *y* directions. The band structure of this system is calculated by means of DFT (see the Supporting Information for details) and shown in Figure 5(a) with black lines, and compared with the band structure of the system without a grain boundary plotted with gray lines. The band gap is reduced considerably by the introduction of a grain boundary, from 592 to 203 meV, which is in qualitative agreement with our tight-binding model and indicates that the used bond modification Δ = 0.15*t*_0_ is reasonable. In order to analyze the character of the bands close to the gap, we integrate the LDOS over the blue and red shaded energy intervals in Figure 5(a) and find that the states below the gap are localized at the grain boundary, while the states above the gap can be attributed to a mixing of bulk and grain boundary states. This system property is confirmed by the average resolved DOS for C(2*p*_*z*_) orbitals in Figure 5(b), which is measured in the bulk (orange curve) and at the grain boundary (green curve), showing that within an energy range of about 500 meV below the gap the states are localized predominately at the grain boundary. We can understand this asymmetry by the fact that the Ti atoms will not only change the carbon bonds but also intrinsically dope the system. This doping occurs homogeneously in the bulk but it does not occur at the grain boundary. We can take into account this doping in our tight-binding model by introducing an on-site potential ε just at the grain boundary. The energy bands in Figure 4(b) are calculated for such a system with ε = 0.3*t*_0_ and show clearly that the solitonic bands (red dashed curves) are moved upward to higher energies while the bulk bands (blue curves) remain unchanged. Therefore, the upper soliton band mixes with the bulk bands, similar to the DFT calculations. Interestingly, we also observe that the band gap is reduced by the on-site potential and the Fermi velocity of the lower soliton band decreases. Note that the unit cell for the tight-binding calculations (∼50 nm) is wider than for the DFT calculations (∼6 nm). In Figure 5 the grain boundary is oriented in the zigzag direction. In the Supporting Information, we demonstrate that the system properties are qualitatively unchanged for a grain boundary in the armchair direction.

**Figure 5:**
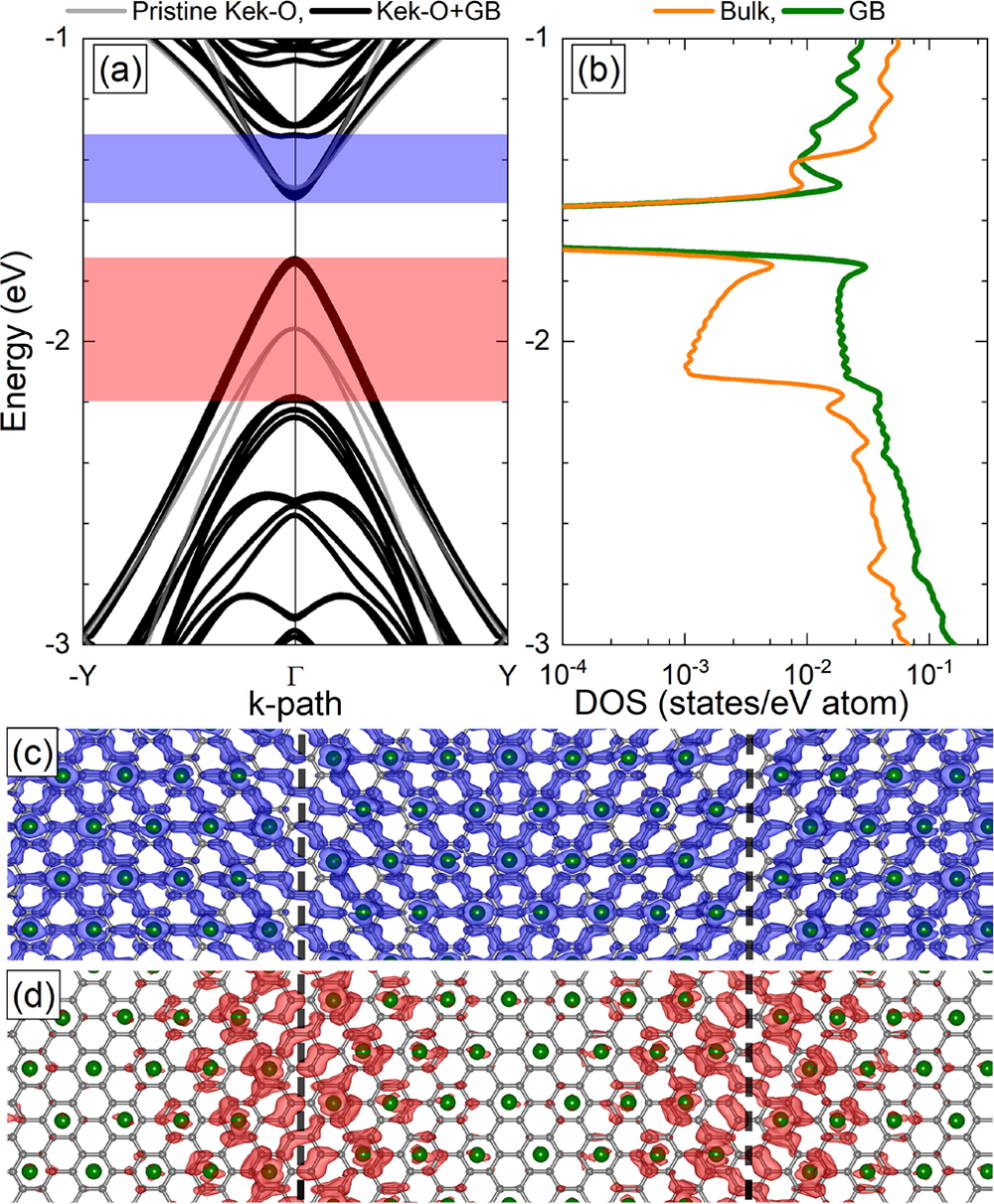
(a) Band structure and (b) average C(2*p*_*z*_) resolved density of states (DOS) for Kekulé-O graphene with a grain boundary. The Fermi energy is set to zero eV. (c) and (d) local density of states (LDOS) calculated using the electronic states enclosed by the blue and red shaded regions in (a). Ti and C atoms are in green and gray color, respectively. The vertical dashed black lines denote the atomic grain boundaries.

Experimentally the atomically precise engineering of Kek-O distortions can be realized by manipulating individual Ti atoms through the tip of a scanning tunneling microscope. Additional DFT calculations (see the Supporting Information) show that the system can be realized alternatively by placing first the Ti atoms on a hexagonal BN substrate and covering it afterward with graphene. Kek-O graphene has been realized successfully by Li intercalations between graphene and a SiC substrate,^29^ which therefore is also a candidate to implement the proposed device. We have selected Ti because its diffusion barrier is higher, making the system thermodynamically more stable. Other atoms like Zr, Rh, Ni, and Ru are also possible candidates for Kek-O engineering in graphene, see the Supporting Information.

In this Letter, we have shown that the current flow in graphene can be guided on atomically thin pathways by means of the engineering of Kekulé-O distortions. A grain boundary in these distortions (see Figure 1) separates the system into two regions and generates a soliton at their interface that transports the current ballistically (see Figure 2). The soliton does not depend on the orientation of the grain boundary with respect to the graphene sublattice and, therefore, can be guided on arbitrary paths through the system (see Figure 3). The soliton is gapped, which permits one to switch efficiently the current flow through electrostatic gates. The existence of the soliton can be understood in terms of a generalized Jackiw–Rebbi model, where the electrons have a positive effective mass in one region and a complex one in the other, which makes our system a platform to study phenomena from high-energy physics. Numerically calculated energy bands agree perfectly with the energy bands from a continuous model of Kek-O graphene and the generalized Jackiw–Rebbi model (Figure 4). Finally, we have demonstrated by means of DFT calculations that the proposed system can be realized by decorations of graphene with Ti atoms (see Figure 5). Technologically, our findings can have important applications in nanoelectronics. In particular, the Kekulé-O engineering in graphene paves the way to nanoelectronic circuits on the scale of individual atoms.

## Supporting Information Available

(a) The Green’s function method for electronic transport, (b) the tight-binding model for Kek-O graphene nanoribbons, (c) the computational methodology of the atomistic model, (d) the robustness of the soliton against defects and imperfections, (e) Kek-O graphene with a grain boundary in the armchair direction, and (f) alternative strategies to realize Kek-O graphene (PDF)
