# Supplementary material for: Atomically Thin Current Pathways in Graphene through Kekulé-O Engineering
Source: Nano Lett. 2024 Feb 8;24(7):2322–7. doi: 10.1021/acs.nanolett.3c04703 (PMC10885192; doi:10.1021/acs.nanolett.3c04703)
Supplement: Supplementary file 1 — nl3c04703_si_001.pdf [file nl3c04703_si_001.pdf]

Supporting Information for the Letter
"Atomically thin current pathways in graphene through Kekulé-O engineering"

Santiago Galván y García,[1,*] Yonatan Betancur-Ocampo,[2,†] Francisco Sánchez-Ochoa,[2,‡] and Thomas Stegmann[1,§]

[1]*Instituto de Ciencias Físicas, Universidad Nacional Autónoma de México, 62210 Cuernavaca, México*
[2]*Instituto de Física, Universidad Nacional Autónoma de México, 04510 Ciudad de México, México*

We present details on (a) the Green's function method for electronic transport, (b) the tight-binding model for Kek-O graphene nanoribbons, (c) the computational methodology of the atomistic model, (d) the robustness of the soliton against defects and imperfections, (e) Kek-O graphene with a grain boundary in the armchair direction and (f) alternative strategies to realize Kek-O graphene.

GREEN'S FUNCTION METHOD FOR ELECTRONIC TRANSPORT

The system properties are studied numerically by means of the Green's function method. As detailed introductions can be found in various textbooks [1, 2], we summarize here only the essential equations. The Green's function of the system is given by

$$G(E) = (E - H - \Sigma_S - \Sigma_D)^{-1}\,, \tag{1}$$

where E is the energy of the injected electrons and H is the tight-binding Hamiltonian, Equation (1). The self-energies $\Sigma_{S/D}$ describe the effect of the contacts on the system and are modeled either by semi-infinite leads or the wideband model, which both are physical legitimate. The wideband model represents a generic metallic contact with a constant surface density of states and its self-energy reads $\Sigma^{\mathrm{wb}} = \sum_{i,j\in\mathrm{contact}} -\mathrm{i}\, t_0 \delta_{ij} \left|i\right\rangle \left\langle j\right|$. Semi-infinite leads are capable to model an infinitely extended structure, where back-scattering at the system edges is absent and the self-energy is given by $\Sigma^{\mathrm{sinf}} = \tau g^{\mathrm{sf}} \tau^{\dagger}$, where g^{sf} is the (recursively calculated) surface Green's function of the semi-infinite lead and τ the matrix which couples it to the central system, see [3] for details.

Finally, the current flowing between the atoms at positions $\boldsymbol{r}_i$ and $\boldsymbol{r}_j$ is calculated by

$$I_{ij} = \mathrm{Im}(t_{ij}\,(G\,\mathrm{Im}(\Sigma)\,G^{\dagger})_{ij}), \tag{2}$$

the local density of states at position $\boldsymbol{r}_i$

$$D_i = \frac{1}{\pi}\left(G\mathrm{Im}(\Sigma)G^{\dagger}\right)_{ii} \tag{3}$$

and the transmission

$$T = 4\mathrm{Tr}\left(G\mathrm{Im}(\Sigma_S)G^{\dagger}\mathrm{Im}(\Sigma_D)\right). \tag{4}$$

TIGHT-BINDING MODEL FOR KEK-O GRAPHENE NANORIBBONS

We consider a Kek-O graphene nanoribbon with zigzag edges, modeled by a nearest-neighbor tight-binding Hamiltonian. Starting with the zigzag edge, the unit cell consists of six atoms, which are linked with the characteristic bonds of the Kekulé-O texture, see Figure 1. The tight-binding Hamiltonian for this chain is very similar to that one of the Su-Schrieffer-Heeger model, but increased three times, and is given by

$$H_{\mathrm{ssh}}(k) = \begin{pmatrix} 0 & 0 & 0 & t^*(k) & t'(k) & 0 \\ 0 & 0 & 0 & 0 & t^*(k) & t(k) \\ 0 & 0 & 0 & t(k) & 0 & t'^*(k) \\ t(k) & 0 & t^*(k) & 0 & 0 & 0 \\ t'^*(k) & t(k) & 0 & 0 & 0 & 0 \\ 0 & t^*(k) & t'(k) & 0 & 0 & 0 \end{pmatrix}, \tag{5}$$

where the functions are defined by $t(k) = te^{i\theta(k)}$, $t'(k) = t'e^{i\theta(k)}$ and $\theta(k) = ka/6$ with the lattice constant a. The regular and distorted carbon bonds are denoted by t and t', respectively. For two coupled zigzag chain we obtain the Hamiltonian

$$H_{\mathrm{2ssh}}(k) = \begin{pmatrix} H_{\mathrm{ssh}}(k) & C \\ C^{\dagger} & H'_{\mathrm{ssh}}(k) \end{pmatrix}, \tag{6}$$

where

$$H'_{\mathrm{ssh}}(k) = \begin{pmatrix} 0 & 0 & 0 & t(k) & 0 & t^*(k) \\ 0 & 0 & 0 & t'^*(k) & t(k) & 0 \\ 0 & 0 & 0 & 0 & t^*(k) & t'(k) \\ t^*(k) & t'(k) & 0 & 0 & 0 & 0 \\ 0 & t^*(k) & t(k) & 0 & 0 & 0 \\ t(k) & 0 & t'^*(k) & 0 & 0 & 0 \end{pmatrix}. \tag{7}$$

is the Hamiltonian of the second chain and the matrix

$$C = \begin{pmatrix} 0 & 0 & 0 & 0 & 0 & 0 \\ 0 & 0 & 0 & 0 & 0 & 0 \\ 0 & 0 & 0 & 0 & 0 & 0 \\ t' & 0 & 0 & 0 & 0 & 0 \\ 0 & t & 0 & 0 & 0 & 0 \\ 0 & 0 & t & 0 & 0 & 0 \end{pmatrix} \tag{8}$$

couples them together.

The tight-binding Hamiltonian of N chains H_{Nssh} consists of a block tridiagonal matrix, whose size depends on

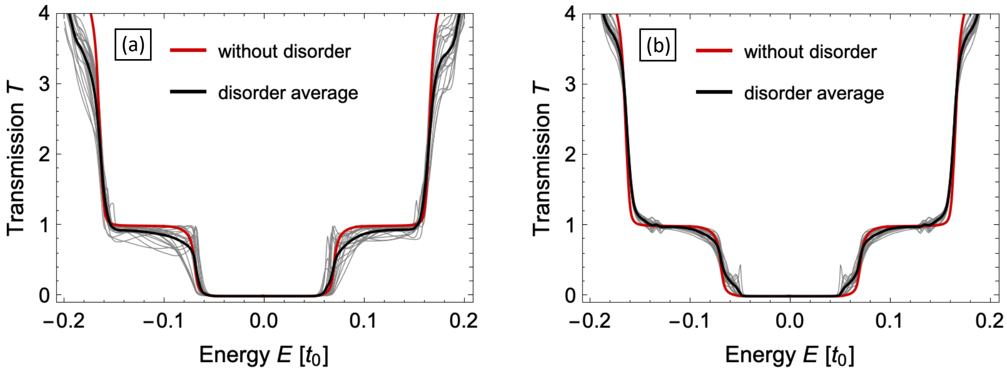

Figure 1. Transmission where a random onsite potential with $\epsilon_{\rm random} = 0.2t_0$ is added (a) and 30% of randomly selected bonds are modified (b). Disorder averages are over 20 realizations (gray curves). The system parameters are otherwise the same as in Figure 2(c) in the main text. The ballistic transmission of the soliton is rather robust.

the width of the homogeneous nanoribbon

$$H_{\rm Nssh}(k) = \begin{pmatrix} H_{\rm ssh}(k) & C & 0 & 0 & 0 & 0 \\ C^\dagger & H'_{\rm ssh}(k) & C' & \cdot & \cdot & \cdot \\ 0 & C'^\dagger & H_{\rm ssh}(k) & C & \cdot & \cdot \\ \cdot & 0 & \cdot & \cdot & \cdot & \cdot \\ \cdot & \cdot & \cdot & \cdot & \cdot & C \\ 0 & 0 & 0 & 0 & C^\dagger & H'_{\rm ssh}(k) \end{pmatrix}. \tag{9}$$

The coupling matrix C' is identical to C in Equation (17), but the sub-diagonal has now the order $\{t, t', t\}$.

In order to build the Hamiltonian of the system with a grain boundary, as shown in Figure 1, it is necessary to consider the specific Kek-O texture in the right region, which consists in changing the bonds in Equation (18). The Hamiltonian reads

$$H_{\rm het}(k) = \begin{pmatrix} H^{\rm left}_{\rm Nssh}(k) & C_0 \\ C_0^\dagger & H^{\rm right}_{\rm Nssh}(k) \end{pmatrix}, \tag{10}$$

where

$$C_0 = \begin{pmatrix} 0 & 0 & 0 & t & 0 & 0 \\ 0 & 0 & 0 & 0 & t' & 0 \\ 0 & 0 & 0 & 0 & 0 & t \\ t' & 0 & 0 & 0 & 0 & 0 \\ 0 & t & 0 & 0 & 0 & 0 \\ 0 & 0 & t & 0 & 0 & 0 \end{pmatrix} \tag{11}$$

couples the two regions periodically. Diagonalizing the Hamiltonian $H_{\rm het}$ in Equation (19), we obtain the electronic band structure shown in Figure 4.

COMPUTATIONAL METHODOLOGY OF THE ATOMISTIC MODEL

We have built a periodic supercell starting from the rectangular unit cell defined in Figure 1 with fifteen unit cells along the armchair direction. This is in order to avoid edge states as in graphene nanoribbons and to study two grain boundaries with the topology in Figure 1 due to periodic conditions. To induce a Kekulé-O distortion, we adsorb Ti atoms on hollow sites of benzene rings. Ti atoms tend to adsorb strongly on hollow sites of graphene monolayer [4].

DFT calculations were performed within the SIESTA code [5, 6]. The electronic states have been expanded using a linear combination of atomic orbitals (LCAO) with a double-ζ plus polarized (DZP) basis-set with a PAO.EnergyShift of 50 meV. Here the valence shell for Ti atoms is $4s$, $3d$ and $4p$, while for C atoms is $2s$, $2p$ and $3d$. The exchange-correlation energy has been treated with the Perdew–Burke-Ernzerhof (PBE) [7] functional within the generalized gradient approximation (GGA) for solids termed as PBEsol [8]. Norm-conserving Trouiller–Martins [9] pseudopotentials were used to describe core-valence electrons interactions. A $1{\times}9{\times}1$ k-grid was used for sampling the reciprocal space with the Monkhorst-Pack scheme [10], and an energy cutoff of 300 Ry for the grid integration of charge density in real space. The electronic temperature was set equal to 0.05 eV with a Methfessel-Paxton statistics. The atomic relaxation was achieved when the inter-atomic forces were $\leq$10 meV/Å, while the electronic relaxation was converged to 10^{-4}. A vacuum gap of 15 Å in the normal (z) direction has been used to prevent interactions between neighbor Ti-doped graphene monolayers in adjacent supercells. Visualization of atomic models and isosurfaces was performed with VESTA program [11].

ROBUSTNESS OF THE SOLITON

Defects and imperfections will naturally arise in the production process of the proposed device. To take into account these and to estimate the robustness of the soli-

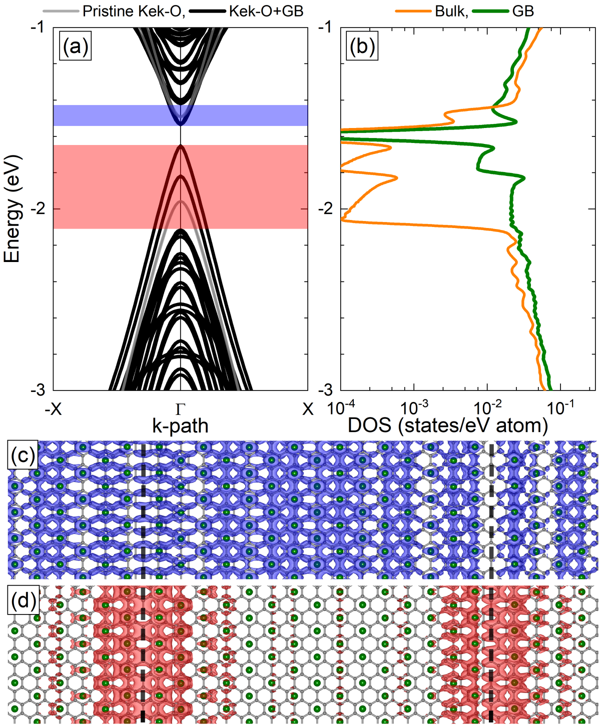

Figure 2. Kek-O graphene with a grain boundary in the armchair direction. The parameters are otherwise identical to Figure 5 in the main text.

ton, we perform additional tight-binding transport calculations. In Figure 1, we show the transmission as in Figure 2(c) in the main text, but in (a) a random onsite disorder potential ($\epsilon_{\mathrm{random}} = 0.2t_0$) is added, while in (b) 30% of the bonds are randomly selected and set to the value $t_0 + \Delta$ in order to model the misplacement of Ti atoms. Disorder averages have been performed over 20 realizations. Both figures demonstrate that the ballistic transmission of the soliton is rather robust as it originates from the topological features of the system.

KEK-O GRAPHENE WITH A GRAIN BOUNDARY IN THE ARMCHAIR DIRECTION

Figure 2 shows the band structure, DOS and LDOS for Kek-O graphene realized by Ti adsorptions. The parameters are the same as in Figure 5 in the main text but the grain boundary is oriented in the armchair direction. The system properties are qualitatively unchanged and thus, prove that the soliton exists independently from the orientation of the graphene sublattice. Note that in Figure 2 and in Figure 5 in the main text, we have used a large rectangular unit cell in the case of a grain boundary (see the LDOS figures), but the smaller rectangular unit cell shown in Figure 1 in the main text is used as reference for the band structure calculation in the pristine case.

ALTERNATIVE STRATEGIES TO REALIZE KEK-O GRAPHENE

In Figure 3, we show band structure and atom-resolved DOS (calculated by DFT) for various types of atoms intercalated between hexagonal BN and graphene. We have selected the elements Ti, Zr, Rh, Ni, and Ru, because the adsorption is in hollow sites with high cohesive energy and high diffusion barrier [4], which favors the stability of the system. Li has been used already experimentally [12] and is added as a reference. Li, Zr and Ti show similar band structure features around $-2\,\mathrm{eV}$ with clear contributions from the carbon atoms. In the case of Ni, Rh and Ru these features are shifted towards $-3\,\mathrm{eV}$. However, for these atoms another band gap opens around the Fermi energy, which may represent another possibility for the Kek-O engineering in graphene (although in these cases the contribution of the C atoms is less pronounced). Note that for the calculation in Figure 3 a small hexagonal unit cell is used, because only pristine systems without a grain boundary are studied.

* santiagogyg@icf.unam.mx
† ybetancur@fisica.unam.mx
‡ fsanchez@fisica.unam.mx
§ stegmann@icf.unam.mx

[1] S. Datta, *Quantum Transport: Atom to Transistor*, 1st ed. (Cambridge University Press, 2005).
[2] M. Di Ventra, *Electrical Transport in Nanoscale Systems*, 1st ed. (Cambridge University Press, 2008).
[3] C. H. Lewenkopf and E. R. Mucciolo, The recursive Green's function method for graphene, Journal of Computational Electronics **12**, 203 (2013).
[4] M. Manadé, F. Viñes, and F. Illas, Transition metal adatoms on graphene: A systematic density functional study, Carbon **95**, 525 (2015).
[5] P. Ordejón, E. Artacho, and J. M. Soler, Self-consistent order-N density-functional calculations for very large systems, Phys. Rev. B **53**, R10441 (1996).
[6] J. M. Soler, E. Artacho, J. D. Gale, A. García, J. Junquera, P. Ordejón, and D. Sánchez-Portal, The SIESTA method for ab initio order-N materials simulation, J. Phys.: Condens. Matter **14**, 2745 (2002).
[7] J. P. Perdew, K. Burke, and M. Ernzerhof, Generalized gradient approximation made simple, Phys. Rev. Lett. **77**, 3865 (1996).
[8] G. I. Csonka, J. P. Perdew, A. Ruzsinszky, P. H. Philipsen, S. Lebègue, J. Paier, O. A. Vydrov, and J. G. Ángyán, Assessing the performance of recent density functionals for bulk solids, Phys. Rev. B **79**, 155107 (2009).
[9] N. Troullier and J. L. Martins, Efficient pseudopotentials for plane-wave calculations, Phys. Rev. B **43**, 1993 (1991).
[10] H. J. Monkhorst and J. D. Pack, Special points for Brillouin-zone integrations, Phys. Rev. B **13**, 5188 (1976).

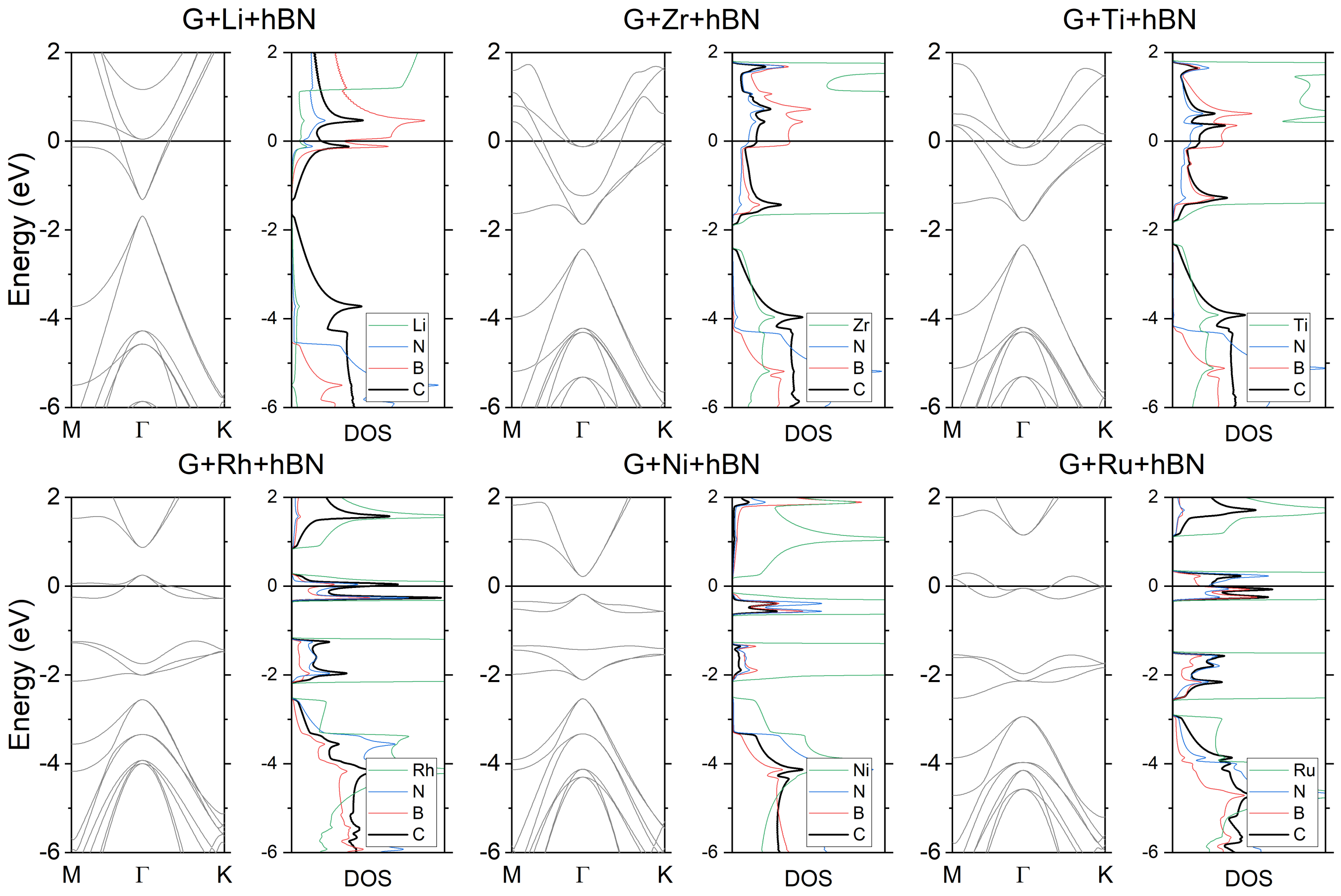

Figure 3. Band structure and atom-resolved DOS for atomic intercalations between hexagonal BN and graphene in order to identify possible candidates for the Kek-O engineering in graphene.

[11] K. Momma and F. Izumi, Vesta 3 for three-dimensional visualization of crystal, volumetric and morphology data, J. Appl. Crystallogr. **44**, 1272 (2011).

[12] C. Bao, H. Zhang, T. Zhang, X. Wu, L. Luo, S. Zhou, Q. Li, Y. Hou, W. Yao, L. Liu, P. Yu, J. Li, W. Duan, H. Yao, Y. Wang, and S. Zhou, Experimental evidence of chiral symmetry breaking in Kekulé-ordered graphene, Phys. Rev. Lett. **126**, 206804 (2021).
